# Addressing cancer invasion and cell motility with quantitative light microscopy

**DOI:** 10.1038/s41598-022-05307-7

**Published:** 2022-01-31

**Authors:** Daniel Zicha

**Affiliations:** 1 CEITEC – Central European Institute of Technology, Brno University of Technology, Purkyňova 656/123, 612 00 Brno, Czech Republic; 2 Institute of Physical Engineering, Faculty of Mechanical Engineering, Brno University of Technology, Technická 2, 616 69 Brno, Czech Republic

**Keywords:** Metastasis, Applied optics

## Abstract

The incidence of death caused by cancer has been increasing worldwide. The growth of cancer cells is not the main problem. The majority of deaths are due to invasion and metastasis, where cancer cells actively spread from primary tumors. Our inbred rat model of spontaneous metastasis revealed dynamic phenotype changes in vitro correlating with the metastatic potential in vivo and led to a discovery of a metastasis suppressor, protein 4.1B, which affects their 2D motility on flat substrates. Subsequently, others confirmed 4.1B as metastasis suppressor using knock-out mice and patient data suggesting mechanism involving apoptosis. There is evidence that 2D motility may be differentially controlled to the 3D situation. Here we show that 4.1B affects cell motility in an invasion assay similarly to the 2D system, further supporting our original hypothesis that the role of 4.1B as metastasis suppressor is primarily mediated by its effect on motility. This is encouraging for the validity of the 2D analysis, and we propose Quantitative Phase Imaging with incoherent light source for rapid and accurate testing of cancer cell motility and growth to be of interest for personalized cancer treatment as illustrated in experiments measuring responses of human adenocarcinoma cells to selected chemotherapeutic drugs.

## Introduction

Cancer poses a major threat to public health worldwide, and incidence rates have increased in most countries since 1990^1^. Cancer in Europe has also been a major burden, with 3.45 million new cases and 1.75 million deaths in 2012^2^. Different types of cancers have not changed their incidence evenly across geographic regions. For example, the incidence of four common cancers in eastern and central European countries (prostate, postmenopausal breast, corpus uteri, and colorectum) started to approach levels in northern and western Europe, where rates were already high^3^. Aggressive cancers increase their incidence generally more dramatically. For example, oropharyngeal cancer significantly increased the incidence from 1983 to 2002 predominantly in economically developed countries^4^. The reasons for this unfavorable situation lie in the limited availability of treatments, inaccuracy of diagnosis, and difficulties with the choice of the most effective treatment. Further research and development in this direction are highly desirable. The growth of cancer cells is not the main problem in aggressive cancers; the majority of deaths are due to invasion and metastasis.

Metastases form by spreading cancer cells from primary tumors to distant parts of the organism. The process, referred to as the metastatic cascade, involves changes in cell morphology and, most importantly, active cell motility with underlying changes in gene expression. The changes are complex since they can be specific for not only different types of cancers relating to the original cancer transformation but also for different groups of patients relating to their genetic background, requiring a personalized approach to the treatment. This situation is difficult to tackle with effective biomedical research.

The introduction section overviews previous findings related to cancer metastasis, which were revealed largely by quantitative imaging of cell behavior leading to the results which focus on the novel application of an invasion assay. Quantitative Phase Imaging has been successfully utilized in a growing number of studies and has been proven as a powerful tool^5,6^. We have finally also used this technology to quantify the motile responses of cancer cells to chemotherapeutic drugs.

### Sarcoma model

**Figure 1:**
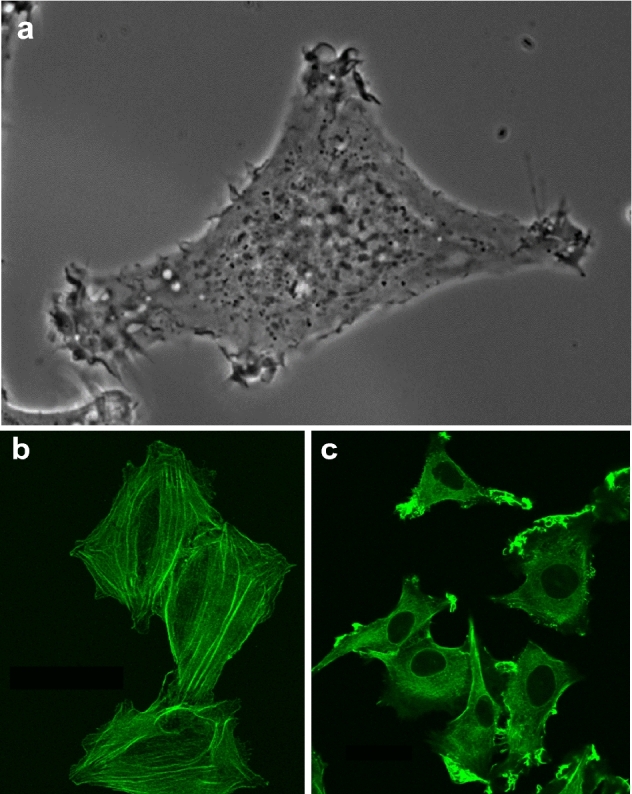
(**a**) One frame from a movie of a sarcoma cell from the metastatic population T15 acquired using LSM 510 (Zeiss) equipped with Plan-Apochromat 63 × /1.4 Oil Ph3. The field size is 93 × 63 µm. (**b**, **c**) Micrographs of fixed sarcoma cells stained with rhodamine-phalloidin acquired as stacks using LSM 510 (Zeiss) are presented as maximum intensity projection with green pseudo-color. (**b**) K2 cells from the non-metastatic line feature conspicuous stress fibers. The field size is 154 × 184 µm. (**c**) A297 cells from the highly metastatic line, which have lost the stress fibers. The field size is 140 × 159 µm.

Our approach has been in using a model with inbred rats LEWIS, which provides a constant genetic background. The model originates from a spontaneously transformed sarcoma K2. The K2 sarcoma cell population spontaneously progressed to a metastatic phenotype producing T15 cell population (Fig. 1a and Supplementary Video S1 online). A further progression resulted in a more-metastatic cell population A297. This concept, with the constant genetic background of the host and one original cancer transformation, is giving us a chance to address the specific mechanisms of the progression to the metastatic phenotype in this model system.

### In vivo

The use of the inbred rat model provides not only the constant genetic background but also a possibility for testing the metastatic potential in vivo. Subcutaneous inoculation of 10^6^ sarcoma cells in suspension gave rise to primary tumors in each animal with all 3 sarcoma populations (K2, T15, and A297). The rats were sacrificed several weeks later, and internal organs were examined for metastasis^7^. None of the 20 rats inoculated with K2 cells had metastasis. With T15 and A297 cells, we detected lung metastasis in 38% of 24 and 90% of 10 rats, respectively. In both populations, T15 and A297, the incidence was increased significantly (χ^2^ P < 0.01) in comparison to the K2 cells.

### Morphology

The established model with the set of related sarcoma populations with different and verified metastatic potential allows following investigation of their in vitro phenotype. Cells in tissue culture were fixed and stained for F-actin with rhodamine-phalloidin. Cells for the K2 population were flat with conspicuous actin stress fibers and limited peripheral activity, while cells from the T15 and A297 populations lost their stress fibers and showed an active periphery (Fig. 1b,c). The decrease of the incidence of cells with F-actin stress fibers in T15 and A297 populations in comparison to the K2 population was statistically significant^7^.

### Motility and chemotaxis

Our main interest was in the dynamic phenotype of the live cells. To investigate not only the speed of cell motility but also their directional migration—chemotaxis, we decided to use the Dunn direct viewing chemotaxis chamber (Weber Scientific)^8,9^. We chose a gradient formed by the introduction of the mixture of PDGF (Platelet Derived Growth Factor) and IGF (Insulin-like Growth Factor) to simulate the situation in the primary tumor, where these gradients would guide the metastatic cells into the bloodstream. Before time-lapse recording in chemotactic gradients, cells were starved in 0.5% serum for 3 h. Overnight time-lapse recording with lapse interval of 5 min used phase-contrast microscopy^7^. The cells were interactively tracked using Kinetic Imaging software, and displacements were analyzed in Mathematica (Wolfram). The 5-min displacements between frames were multiplied by 12 to get the speed in µm h^−1^. K2 cells achieved mean speed ± SEM of 11.1 ± 0.5 µm h^−1^ (N = 171). In comparison to the K2 cells, the T15 and A297 cells had significantly increased speed ± SEM of 17.0 ± 0.7 (N = 116, ANOVA P < 0.05) and 19.0 ± 0.9 (N = 176, ANOVA P < 0.01), respectively. In conclusion, the cells from populations with significantly increased metastatic potential lacking F-actin stress fibers also migrated with significantly increased speed. In order to evaluate chemotaxis, the directional movement of cells, we used statistics of directional data^10^. Therefore, we needed to convert the trajectories into directions, and we achieved that by determining the directions of cells on reaching a 70 µm horizon distance from the starting points of their trajectories. Only a subset of the cells reached the horizon (61 of 171 K2 cells, 81 of 116 T15 cells, and 86 of 176 A297 cells), and that is a fair system since chemotaxis is not defined for cells, which are not motile. The Rayleigh test revealed significant unimodal clustering (P < 0.01) of directions only in the cells from the metastatic lines, T15 and A297. We concluded that the T15 and A297 cells showed chemotaxis since the mean direction coincided with the direction of the increasing concentration of PDGF/IGF in the gradient. More precisely, the direction of the gradient was within the 95% confidence interval of the mean directions. The statistics of directional data does not provide means for testing differences between populations. Therefore we used ANOVA (Analysis of Variance) with unbalanced data and hierarchical model^11^ for displacement components in the direction of the gradient and found that only the A297 cells showed significantly stronger chemotaxis than the K2 cells (P < 0.05).

### Rapid actin transport

The actomyosin machinery is instrumental in the motile responses of cells. To explore actin dynamics in the T15 cells, we developed a method of FLAP (Fluorescence Localization After Photobleaching) and showed that actin monomer was actively transported into the protrusion areas in the rapidly moving T15 cells from the metastatic population (see Supplementary Fig. S2 online). This active transport is mediated by myosin since it is abrogated by myosin light chain kinase inhibitor ML7. Therefore, we concluded that the active transport is a result of the contractile forces in the T15 cells^12^.

### Invasion assay

At the target place in the metastatic cascade mediated by the hematogenous pathway, the cancer cells have to extravasate from the bloodstream into the surrounding tissue crossing the blood vessel wall. The inner surface of the blood vessel is formed by a layer of endothelial cells. We decided to investigate this step in vitro using monolayers of rat brain endothelial cells (RBE4), observing the details of the invasion of the sarcoma cells^13^. We never observed the non-metastatic K2 cells crossing the monolayer while the T15 cells invaded the monolayer readily. The invasion assay was developed to allow observation under flow conditions, which is the case in a real blood vessel situation. We found that the flow condition initially enhanced the invasion of the T15 cells after they adhered to the endothelial monolayer. In the experiments, without any flow, the invasion became faster only at a later time in the experiment. We hypothesized that the mechanical stimuli of the flow activate the invasion initially when the cell contractile forces are dominant. While later, the more efficient invasion without flow is dominated by the release of proteolytic enzymes, which is not effective under the flow conditions.

### Gene expression and metastasis suppressor 4.1B

Once we characterized the populations from the sarcoma model and established the in vitro phenotype correlating with their metastatic potential in vivo, we were ready to examine their underlying gene expression profiles. We used Affymetrix Rat 230A GeneChips with 15,924 probes testing for 10,972 genes, which is a substantial subset of the total ~ 25,000 genes in rats^7^. Due to the straightforward availability of the inbred rats, we decided to use cells from the primary tumors as well as cells from tissue culture. The aim was in revealing not only the differences associated with the tumor progression towards the metastatic phenotype but also the differences between the *in vivo* and *in vitro* situations. The choice of the primary tumor rather than metastasis as the source of the in vivo material might be seen surprising since in experiments based on repeated intravenous injections of cells from metastases produced more efficient formation of new metastases^14^. With the intravenous injections, however, the selection procedure focuses only on the part of the metastatic cascade—extravasation and homing. In our experiments, when we passaged cells directly from one animal to another and found that subcutaneously introduced fragments from primary tumors gave rise to more metastasis than fragments taken from metastases. A plausible explanation is that the complete metastatic cascade, tested by spontaneous metastasis shed from primary tumors, is a much more complex process involving subsequent switches from proliferation to migration and back again. This switching machinery is fragile and is not readily preserved after it has been used and the cells in the primary tumor are better prepared for the formation of metastases.

The expression results are summarized in Supplementary Fig. S3, presenting previously unpublished heatmaps of relative expressions calculated from normalized expression levels. The genes in the heatmap were arranged using cluster analysis. The dominance of the blue color representing downregulation of gene expression shows that the progression towards the metastatic phenotype is linked to further dedifferentiation. It is also noticeable that not all genes are regulated similarly in vitro and in vivo. This observation shows that results with established cell lines based on in vitro data only need to be taken with caution.

More reduced information is also in Supplementary Fig. S3, presenting only significant changes larger than 2.5 ×. The statistical significance was corrected by the Benjamini–Hochberg algorithm^15^ to avoid excess of false-positive calls. With these selection conditions, we reduced the ~ 11,000 tested genes to 23 and believe that it is largely due to the power of the inbred rat model with the related cell populations only changed in their metastatic potential. We propose that this list contains the drives of the phenotype changes in this case. We confirmed the results for 10 of the genes with RT-PCR, cloned 4 of them, and when we started to obtain interesting results with Epb41l3 gene coding for protein 4.1B, we fully focused on it and identified it as a novel metastasis suppressor.

The amount of the 4.1B protein in the K2 cells, in vivo and in vitro, is similar to proteins expressed at an average level in the cells. The levels of 4.1B in the T15 cells, as well as the A297 cells, are significantly reduced 37 × in both situations. We found that its experimental suppression by microinjection of Rat epb41l3 siRNA led to the disappearance of the F-actin stress fibers in K2 cells, which makes their morphology resemble the more metastatic phenotype. Only a rescue experiment provides unequivocal proof that the 4.1B downregulation is responsible for the disappearance of the stress fibers. We were not able to perform the rescue experiment in the fragile sarcoma cells since they would not survive 2 rounds of chemical transfection. But we were successful in HeLa cells. Human epb41l3 siRNA suppressed the F-actin stress fibers in the Hela cells significantly, and subsequent transfection with a resistant 4.1B construct returned the incidence of stress fibers to normal levels. This result demonstrates that the role of 4.1B protein is more general and also valid in human carcinoma cells.

Dynamic experiments with the Dunn chamber revealed that 4.1B affected motility and chemotaxis as well as morphology. Cells were seeded on diamond scratched coverslips for easy localization and microinjected with expression constructs or siRNA. With each microinjection, we coinjected the EGFP (Enhanced Green Fluorescent Protein) expression construct to identify successfully injected cells by fluorescence. Supplementary Video S4 illustrates the procedure. The 4.1B siRNA in non-metastatic K2 cells significantly enhanced cell speed to about double the rate. Conversely, the exogenous expression of 4.1B in the metastatic T15 cells reduced speed to about half of the wild-type cells, and their chemotaxis was also abrogated.

Microscopy observations with the 4.1B-EGFP fusion protein expressed in K2 and HeLa cells revealed that the protein was localized in mature focal adhesions and enriched in membranes, as shown before. Taking all the evidence together, we produced a hypothesis about the mechanism of the role of 4.1B as a metastasis suppressor enhancing cell motility. The 4.1B protein stabilizes the focal adhesions and facilitates the anchorage of F-actin stress fibers in the focal adhesions. These relatively stable structures act as a brake to cell motility. In the metastatic cell populations, the 4.1B protein is eliminated, the stress fibers cannot be anchored in the focal adhesions and disappear. The cells are now more mobile and can turn more easily to orient themselves in the chemotactic gradients and navigate through the metastatic cascade.

## Results

### Role of 4.1B in the invasion

After we published the evidence for protein 4.1B acting as a metastasis suppressor^7^, the laboratory of Richard Hynes confirmed the findings using knock-out mice and patient data^16^. They proposed that the mechanism may be mediated by apoptosis. The migration in 2D is not always controlled similarly to the 3D situation^17^, although there are situations where it is controlled the same way, such as the dorsal closure in vivo and mink lung cells in 2D tissue culture where TGFβ1 induces increased motility and spreading in both situations^18^. Here we present experiments exploring the role of 4.1B in our invasion assay developed earlier^13^. The fragile sarcoma cells do not survive the chemical transfection required for these experiments. Therefore, we used non-small cell lung carcinoma cells A549 with pneumocytes type II, both of human origin.

**Figure 2:**
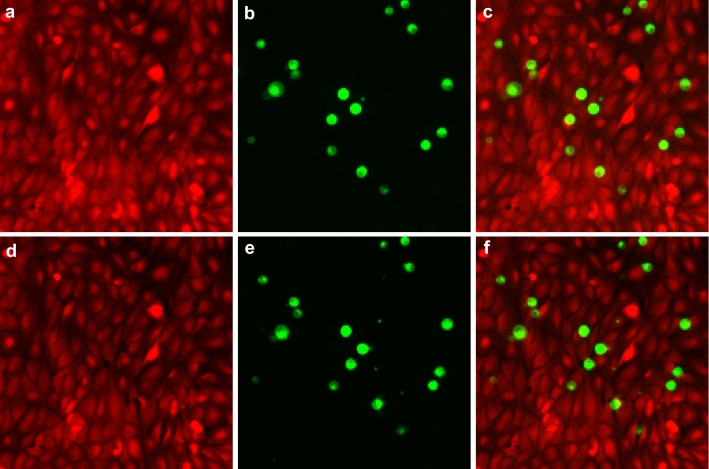
Images from a time-lapse sequence recording red and green fluorescence of pneumocytes type II and human non-small cell lung carcinoma A549 cells, respectively. (**a**) pneumocytes labeled red at time point 0 min; (**b**) A549 cells labeled green at time point 0 min; (**c**) overlay at time point 0 min; (**d**) pneumocytes labeled red at time point 210 min; (**e**) A549 cells labeled green at time point 210 min; (**f**) overlay at time point 210 min. The yellow arrow at the same position in each image points to an invasion even. The green A549 cell migrated towards the arrow first and subsequently invaded the confluent pneumocyte monolayer opening a hole. This created a black gap between the red pneumocyte cells, which were originally in close contact. We know from confocal microscopy that the invading cells attach to the substrate when displacing the pneumocytes. The presented field size is 434 × 434 µm.

Exogenous expression of 4.1B protein in the A549 cells significantly abrogated their invasion (Table 1, Fig. 2 and Supplementary Video S5). This further confirms the role of 4.1B as the metastasis suppressor affecting cell motility. Control transfection with an empty vector achieved 21% of invading cells, and with 4.1B the fraction was reduced to only 3%. Suppression of the residual 4.1B by siRNA produced a moderately higher number of invading cells (26%), but the increase was not significant. This is not surprising because the A549 cells express only low 4.1B levels (https://www.ebi.ac.uk/gxa/experiments/E-GEOD-38332/Downloads) being a model of aggressive metastatic cancer.

**Table 1:** Results of the assay with A549 cells invading monolayers of pneumocytes type II.

Treatment	The fraction of invading cells Mean ± SEM	Number of movies	Total number of cells	ANOVA P-value
Empty vector	0.207 ± 0.050	17	1616	Reference
4.1B protein	0.031 ± 0.006	17	1361	< 0.5
4.1B siRNA	0.255 ± 0.042	7	622	n.s

Protein 4.1B significantly suppressed the invasion. Downregulation of 4.1B achieved a numerically higher mean value but without statistical significance.

### Incoherent-light-source quantitative phase imaging (iQPI)

**Figure 3:**
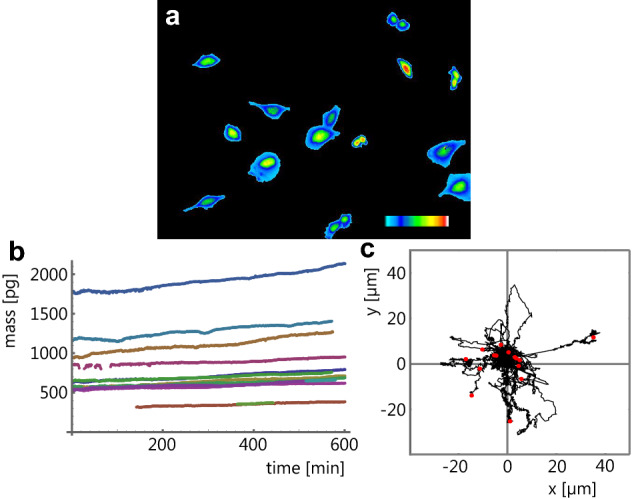
(**a**) A frame from a time-lapse recording of cells from the metastatic T15 sarcoma population using a Horn type Leitz double beam transmitted-light interference microscope equipped with a 20 × twin objective lens and an Orca ER camera (Hamamatsu). The field size is 634 × 483 µm. The pseudo-color bar represents a range of dry-mass densities from 0 to 2.1 pg µm^−2^. (**b**) Growth curves of individual cells and clusters of cells from the recording. (**c**) Rose plot of trajectories of mass centroids of cells from the recording. Each trajectory was positioned with its starting point in the origin, and the endpoint is marked with a red dot.

The invasion assay with the exogenous expression of the 4.1B protein confirmed similar results to the previous motility observations in 2D tissue culture. The presence of the protein suppressed the invasion in cancer-transformed cells as well as their 2D motility. It is therefore obvious that the 2D observations can provide useful information. In order to improve the quality of the 2D observations, we introduced the iQPI with a double-beam configuration. This arrangement provides accurate measurements of small details in cells since it does not suffer from coherence speckles and other artifacts as described by Chmelik^19^. Example imaging from this microscopy is illustrated in Fig. 3a and Supplementary Video S6. The image is calibrated for dry-mass density in pg µm^−2^ (pg, picogram, is 10^−12^ g) and presented as a 2D map.

The integrated signal inside a cell thus reveals the total dry-mass of the cell, and its growth (increase in dry-mass) can be evaluated as a function of time (Fig. 3b). Exponential interpolation of these growth curves gives dry-mass doubling time. The mean dry-mass of the cells ± SEM was 802 ± 110 pg and mean dry-mass doubling time ± SEM was 36.2 ± 11.7 h. In cells stably growing in tissue culture, the dry mass doubling time is equivalent to the cell cycle duration and can be determined much more rapidly.

The iQPI can measure accurately and rapidly not only the growth of cells but also their motility. In subconfluent cultures, it is feasible to evaluate translocations of centroids of the cell’s dry-mass and obtain accurate cell trajectories (Fig. 3c). The plot represents 14 cells with 7173 displacements achieving a mean speed ± SEM of 33.19 ± 2.00 µm h^−1^.

**Figure 4:**
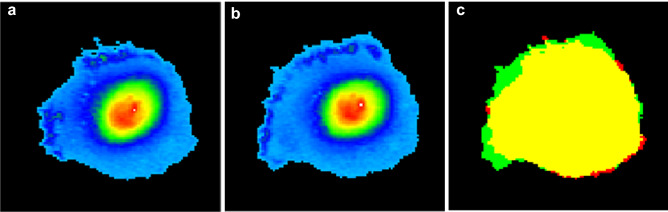
Illustration of protrusions and retractions for one cell from movie related to Fig. 3. (**a**) The cell at time-point 371 min; (**b**) the same cell at time point 381 min and (**c**) superimposed outlines with the protrusion in green and retraction in red. The field size is 128 × 135 µm. The common area occupied in both frames is in yellow. The green protrusion is the newly allocated area in the frame (**b**), and the red retraction is the withdrawn area allocated in the frame (**a**), which is no longer allocated in (**b**).

More confluent cultures, however, do not produce many accurate trajectories, but the iQPI can still produce a useful measure of cell motility in terms of protrusion and retraction^20^ illustrated in Fig. 4. All the cells in the movie achieved mean 1-min cell protrusion dry-mass ± SEM of 2.657 ± 0.808 pg covering mean 1-min cell protrusion area ± SEM of 71.28 ± 8.95 µm^2^. The mere increase in the size of protrusions is not sufficient for effective motility. The protrusion process in cells has to achieve asymmetry, which can be measured by dynamic polarity, defined as the distance between protrusion centroid and retraction centroid. The mean dynamic polarity ± SEM in this movie was 9.902 ± 0.620 µm.

### Responses of A549 cells to chemotherapeutic drugs

**Figure 5:**
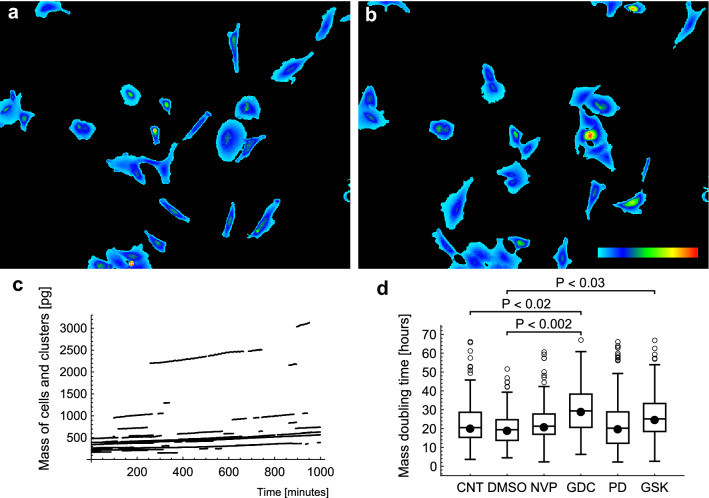
Example images from a time-lapse recording with control A549 cells: (**a**) time point 0 h and (**b**) time point 10 h. The field size is 634 × 483 µm. The pseudo-color bar represents a range of dry-mass densities from 0 to 2.7 pg µm^−2^. (**c**) Growth curves illustrate the increase in dry-mass of cells and clusters in the recording. (**d**) Box and whisker plot of the mass doubling time showing a significant reduction in growth—extended doubling time, with GDC in comparison to CNT and DMSO. GSK achieved a smaller reduction, only significant in comparison to DMSO. Dots represent medians, boxes span 50% of the data, whiskers span the full range of data, and outliers are shown as open circles.

**Figure 6:**
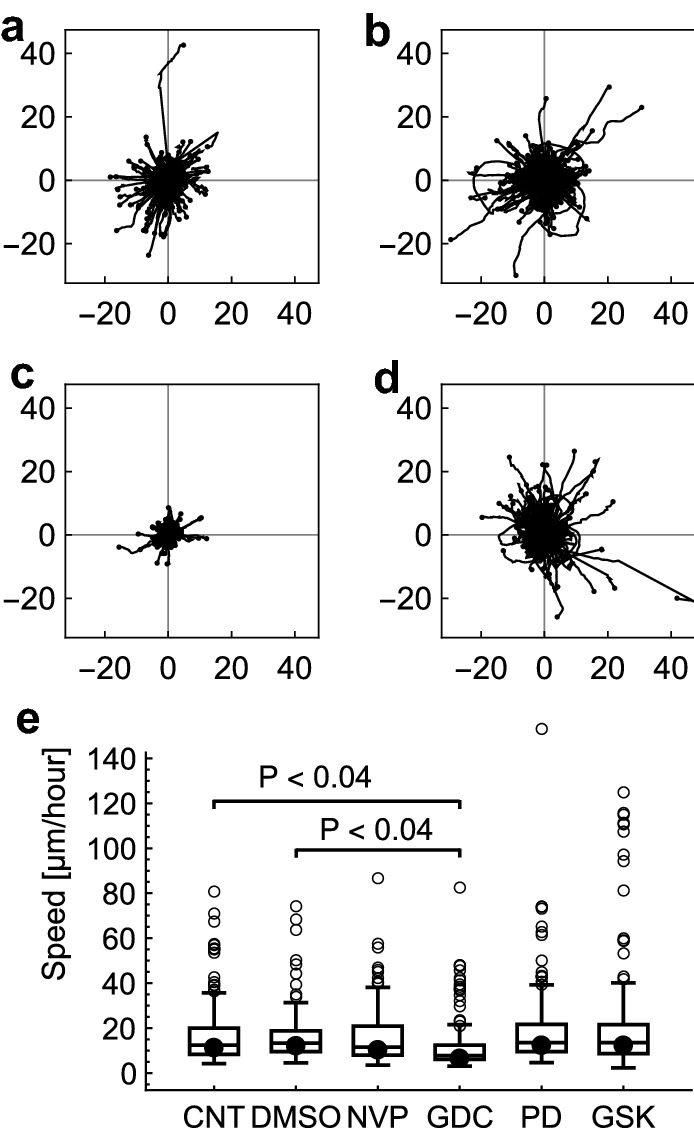
Rose plots of 30-min cell trajectories: (**a**) CNT, (**b**) DMSO, (**c**) GDC and (**d**) GSK. The starting points of the trajectories were shifted to a common origin. Axes are in µm. (**e**) Box and whisker plot of speed. Only GDC achieved a significant reduction of cell motility in comparison to both controls, CNT and DMSO. The plot has the same style as in Fig. 5d.

The iQPI procedure was used to measure responses of A549 cells to selected chemotherapeutic drugs in order to demonstrate the potential of pretesting drugs with patient biopsy cells. Four drugs were used: an IGF1R inhibitor NVP-AEW541 (NVP), a PI3K inhibitor GDC0941 (GDC), a MEK inhibitor PD0325901 (PD), and another MEK inhibitor GSK1120212 (GSK). These drugs were previously used, and their specificity was tested by Julian Downward laboratory, Cancer Research UK London Research Institute^21^. Example images from a time-lapse recording with control medium (see Supplementary Video S7 online) are in Fig. 5a,b. Dry-mass increase of individual cells and cell clusters was significantly suppressed by GDC and less so by GSK (Fig. 5c,d, and Supplementary Video S8, S9). Speed of cell translocation was significantly reduced only by GDC, as illustrated by rose plots in Fig. 6a–d and the box and whisker plot in Fig. 6e. The speed suppression can be mediated by a reduction in of cell protrusion or dynamic polarity of protrusion/ retraction. In this case, the speed was suppressed mainly by a significant reduction of the dynamic cell polarity (ANOVA P < 0.03). The mean dynamic polarity ± S.E.M. of 17.2 ± 0.8 µm (N = 27 707) and 16.9 ± 1.2 (N = 27 135) was achieved by CNT cells, and GDC treated cells, respectively. There were no significant changes in total protrusion mass, but the maximum mass value in the protrusion area was significantly reduced by the GDC treatment from 85 ± 2 femtogram (femtogram is 10^−15^ g) for CNT cells to 69 ± 2 femtogram, ANOVA P < 0.05. Overview of all mean values ± S.E.M. and statistical data are in Supplementary Table S10 online. GDC was thus identified as the best drug from the set for the A549 cells, significantly reducing growth as well as the speed by reducing dynamic polarity and maximum mass density in the protrusion area. The GSK drug only partially reduced the growth with no effect on speed. The other two drugs NVP and PD did not achieve any significant changes in this test.

## Discussion

The use of an inbred rat model with spontaneously transformed sarcoma cell populations at varying levels of tumor progression towards the metastatic phenotype allowed us to establish correlations between their metastatic potential in vivo and cell behavior in vitro. The cells from metastatic populations lost conspicuous F-actin stress fibers, increased speed of cell motility in 2D tissue culture, and showed significantly enhanced chemotaxis in gradients of PDGF/IGF. Fluorescence Localisation After Photobleaching showed that active transport of monomeric actin into the protrusion areas was instrumental in the fast motility of the sarcoma cells from the metastatic populations. In addition, the non-metastatic cells were not able to invade monolayers of endothelial cells while the metastatic cells invaded readily. Gene expression analysis identified protein 4.1B as a novel metastasis suppressor, which is lost in the metastatic cell populations. This loss induces the abrogation of F-actin stress fibers and is responsible for increased motility and chemotaxis of the cells from the metastatic populations.

It was previously known that the 4.1B protein is often downregulated in cancers and scientists found it surprising that 4.1B knock-out mice did not have an increased incidence of spontaneous cancers. After our 4.1B publication, Richard Hynes laboratory confirmed that the 4.1B is a metastasis suppressor and not a cancer suppressor by inducing cancers in the 4.1B knock-out mice and observed more invasion and metastasis. They also used patient data. It is therefore obvious that knock-out experiments on their own are limited in detecting subtle molecular mechanisms. The lack of the metastasis suppressor 4.1B manifests itself only in the progression towards the metastatic phenotype and can be efficiently identified by the fairly complex procedure presented in this manuscript. Subsequently, it is easier to confirm in another context, such as patient data, which tend to be more heterogeneous.

Here we show for the first time that the downregulation of the metastasis suppressor 4.1B is also responsible for invasion using experiments where transfected A549 cells invade monolayers of pneumocytes type II. The invasion test was first developed for the rat sarcoma cells invading more relevant rat endothelial monolayers, but we found that the human A549 carcinoma cells performed equally well with the epithelial pneumocytes, and we obtained meaningful results. These results further confirm our originally proposed hypothesis that the mechanism of the metastasis suppressor 4.1B is in its effect on cell motility and that our original 2D observations, including the localization of 4.1B in focal adhesions, were pointing in the correct direction.

Therefore, we started focusing on the use of iQPI, which is emerging label-free microscopy^22^, to obtain more accurate information on 2D motile processes rapidly, which is important particularly in metastasis research^23^. Similar approaches have been published. Vasilenko et al. used laser-based QPI to address the morphology of circulating tumor cells in relationship to their metastatic potential^24^. We were more interested in drug screening and Murray et al.^25^ and Mir et al.^26^ successfully used QPI exactly for that purpose but only evaluated cell growth. As our biological results indicate, our main interest was in the motility of cancer cells, and Frame et al.^27^ evaluated translocation of cancer cells using ptychography phase measurements, and Hellesvik et al.^28^ used the coherent laser-based Holomonitor system for 3D tracking of osteosarcoma cells.

With our biological model, including the metastasis suppressor, the presented results demonstrate a clear relationship between cancer cell behavior in vivo, in 3D and in 2D assays. This is a justification for using accurate double-beam iQPI for 2D analysis, and we demonstrate significant changes with drug responses based not only on translocation but on protrusion/ retraction measurements as well. These are of special importance in metastasis research since cells tend to form clusters, and translocation of individual cells cannot be measured as accurately under these circumstances.

Here we present data on the T1 sarcoma cells from the metastatic population. We also demonstrated the suitability of this approach for pretesting chemotherapeutic drugs by measuring responses of A549 cells to 4 selected treatments. One of the drugs, GDC, achieved the best performance in significant suppression of both, cell growth and motility. The measurements of growth rely on a dry-mass increase in cells, which can produce reliable results even if we do not encounter many divisions of cells, which are used in more traditional ways of evaluating cell growth. Cell translocation is measured very accurately, and even shorter observation times, such as 4 hours, would produce similar conclusions. The speed reduction can be mediated by changes in protrusion mass or dynamic polarity of protrusion/ retraction. In the case of the GDC drug, the speed of cell motility was decreased by reduced dynamic polarity, while the total mass of cell protrusion was not significantly changed. The only maximum value of mass in the protrusion area was reduced. This information is useful for potential further investigations of specific changes in cytoskeleton re-organization involved in the mechanism of the action of this drug.

The Horn design Leitz microscope has a fundamental setback since it is no longer commercially available. Moreover, it can only be used for one observation field at a time and does not combine with fluorescence. All these setbacks are resolved in a commercially available Q-Phase Microscope from Telight, which produces images comparable to the Horn design Leitz microscope since it is also a double-beam interference microscope with incoherent light source producing phase imaging which is free of coherence speckles and other artifacts. The Q-Phase operates in a holographic mode, and the phase-shifting procedure is not necessary. The optical thickness of cells is calculated from the holographic fringes using Fourier transforms. The exposure time is thus reduced to one camera frame, and faster processes can be observed. The Q-Phase implementation of iQPI features coherence gating and thus offers not only accurate quantitation of 2D cell motility but also qualitative analysis in 3D environments such as collagen gel^29^. The Q-Phase has been proven as a valuable tool for the characterization of primary cancer cells^30^, the distinction of different types of cancer cell death^31,32^, and studying oxidative stress resistance in metastatic prostate cancer^33^. The microscope is also equipped with an accurate multi-position stage and fluorescence. The Q-Phase is thus suitable for further research in cancer cell biology and has the potential of contributing to personalized cancer treatment.

## Methods

### Cell culture and labeling

Regularly passaged pneumocytes type II and A549 cells were cultured in Dulbecco’s modified Eagle’s medium (DMEM) with 10% fetal bovine serum and kept at 37 °C in a humidified atmosphere enriched with 5% CO_2_.

Pneumocytes type II seeded in 12-well plates were labeled by a specially developed procedure^13^. Briefly, CellTracker Orange CMTMR (Invitrogen Ltd) was diluted to 100 μM concentration in DMSO (Sigma) and kept at – 20 °C for at least 2 weeks before use. We found that without the unusually high concentration and long incubation at − 20 °C, the plasma membrane labeling internalizes and photo-bleaches rapidly. The confluent cells were labeled with 1.5 μM concentration for 5 min at 4 °C and subsequently washed.

For the invasion assay, the A549 cells close to confluency in vented flasks were labeled with 10 μM green Vybrant DiO solution (Invitrogen, USA) by a 15 min incubation at 37 °C and subsequently washed, released by brief exposure to trypsin versene solution (1:5) and placed above the labeled pneumocytes into the 12-well plates ready for microscopy.

The T15 sarcoma cells^34,35^ were maintained in MEM with Hank’s salts supplemented with 10% bovine serum (SML, Germany), 0.09% sodium bicarbonate, 0.12 g/l Na-pyruvate (Sigma), and 1 mM glutamine, at 37 °C with 5% CO_2_.

For the iQPI recording, the T15 or A549 cells were seeded on coverslips and placed over a chamber slide consisting of a drilled through slide with a coverslip permanently glued to the bottom side of the slide with Sylgard 184 (Dow Corning) and filled with medium with or without chemotherapeutic drugs. The top coverslip with cells on the underside was sealed on with a wax mixture made from beeswax (Fisher), soft yellow paraffin (Fisher), and paraffin wax (melting point 46 °C; Fisher) in the ratio 1:1:1.

### Invasion assay

The invasion assay is illustrated in Supplementary Fig. S11 online, showing a 3D reconstruction of an adherent and an invading cell. The invasion assay time-lapse acquiring alternating fluorescence images in red and green channels with 15-min lapse interval was performed for 210 min with Metamorph software (Universal Imaging) and inverted ECLIPSE TE2000-E Microscope (Nikon), which was equipped with 10 ×/0.3 objective lens and iXonEM+ DU-888 back-illuminated EMCCD scientific camera (Andor). The microscope was placed in a 37 °C environmental incubator with the delivery of humidified CO_2_ atmosphere (Life Imaging Services). The full observation field recorded by the camera was 1308 × 1308 μm.

The acquired invasion assay time-lapse recordings were analyzed by automatic image processing. The A549 cells were segmented and tracked as green objects. The fraction of invading A549 cells was determined by their coincidence with openings in the pneumocyte monolayer detected by a significant reduction of the red fluorescence signal at the same location as illustrated in Fig. 2. The performance of the procedure was manually validated.

### iQPI

Our iQPI was achieved by a double beam transmitted-light interference Leitz microscope (Horn design) and was based on the previously developed DRIMAPS system^36^. We adapted the DRIMAPS system by newly introducing stable LED illumination, Hamamatsu Orca ER CCD camera with high dynamic range and easy-to-upgrade Micro-manager^37^ control. The illumination beam from the 530 nm LED is separated on a beam splitter, and the two parts progress through 2 identical microscopes in one housing. Only one of the beams, the object beam, passes through cells and the other, the reference beam, travels through a dummy chamber without cells. The two beams are recombined in front of a Hamamatsu Orca ER CCD camera where they interfere and the optical thickness of cells, having introduced a phase shift in the object beam, results in intensity changes on the camera chip. These intensity changes depend on the optical thickness of cells sinusoidally. To achieve linear measurements of the cell thickness, we use a phase-shifting procedure, which introduces an additional overall phase shift by a motorized optical wedge in the reference beam. The movement of the optical wedge is accurately synchronized with fast image acquisition under computer control using script in Micro-Manager. Four frames are acquired at each time point in the time-lapse sequence with a shift of π/4. The Optical Path Difference (OPD) is then simply calculated as the arctangent of a ratio of 2 image differences^38^. The OPD introduced by cells depends on the amount of dry-material in the cells and its specific refractive index. Since the specific refractive indices of all main components of cells are similar, the OPD can be expressed as dry mass density using an average specific refractive index^39^. Our image processing was based on a previously developed system for analysis of subconfluent cells^40^ and was further improved in Mathematica (Wolfram) and C. The system includes removal of background fluctuations, background reconstruction and subtraction. The background reconstruction uses the most frequent phase values in time. Areas covered by cells during the entire time-lapse do not feature dominant phase values and are interpolated by a novel algorithm utilizing local values in the periphery of these areas and more global values in their centers, ensuring smooth features of the reconstructed background. An example of a quantitative phase image before thresholding is presented in Supplementary Fig. S12 online and demonstrates clean background allowing thresholding level around 1% of the maximum cell signal preserving details in the cell periphery. Subsequent cell segmentation newly allows the segmented cells to contain negative phase values, which is important to preserve accuracy in the thinnest parts of the cells. The segmentation is followed by automatic cell tracking based on the nearest object with a similar mass in the next frame and calculation of morphometric parameters, protrusions, and retractions.

## Supplementary Information

Supplementary Information 1.

Supplementary Video S1.

Supplementary Video S4.

Supplementary Video S5.

Supplementary Video S6.

Supplementary Video S7.

Supplementary Video S8.

Supplementary Video S9.

## Data Availability

The datasets generated during and/or analysed during the current study are available from the corresponding author on reasonable request.
