# Supplementary material for: Addressing cancer invasion and cell motility with quantitative light microscopy
Source: Sci Rep. 2022 Jan 31;12:1621. doi: 10.1038/s41598-022-05307-7 (PMC8803927; doi:10.1038/s41598-022-05307-7)
Supplement: Supplementary file 1 — Supplementary Information 1. [file 41598_2022_5307_MOESM1_ESM.pdf]

Supplementary Information for

Addressing cancer invasion and cell motility with quantitative light microscopy

Daniel Zicha[1,2,*]

[1]CEITEC – Central European Institute of Technology, Brno University of Technology, Purkyňova 656/123, 612 00 Brno, Czech Republic
[2]Institute of Physical Engineering, Faculty of Mechanical Engineering, Brno University of Technology, Technická 2, 616 69 Brno, Czech Republic
*daniel.zicha@ceitec.cz

Supplementary Video S1 Time-lapse sequence of a T15 cell (related to Fig. 1a) acquired with a lapse interval of 1.4 s.

Supplementary Figure S2

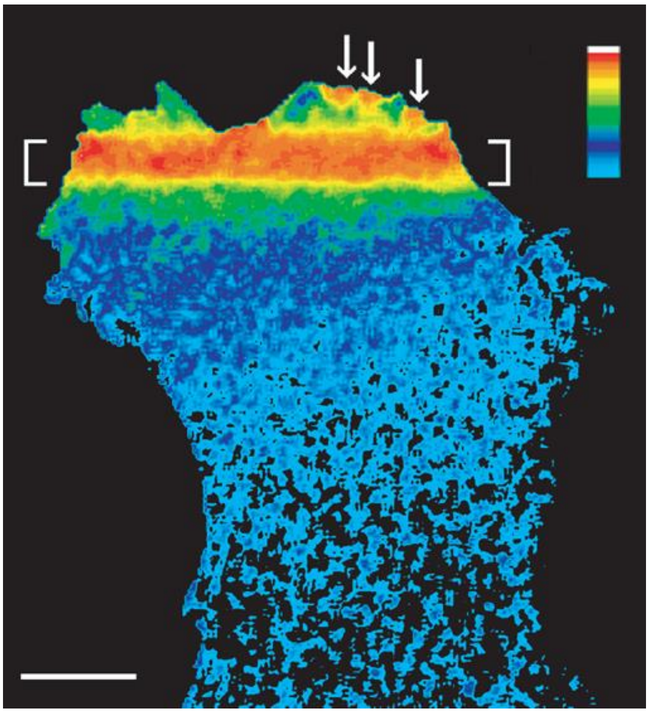

Image of the front part of a metastatic T15 cell, crawling towards the top, representing the distribution of the fraction of the delivered actin molecules 5 s after FLAP-labeling in the region between the white brackets. We calculated the image from a single optical section acquired in two fluorescence channels with an LSM 510 (Zeiss) equipped with a 63×/1.4 objective lens. Arrows mark regions resulting from the rapid active transport. The scale bar represents 5 µm and the pseudo-color bar indicates delivered actin fractions between 0.0 and 0.9.

Supplementary Figure S3

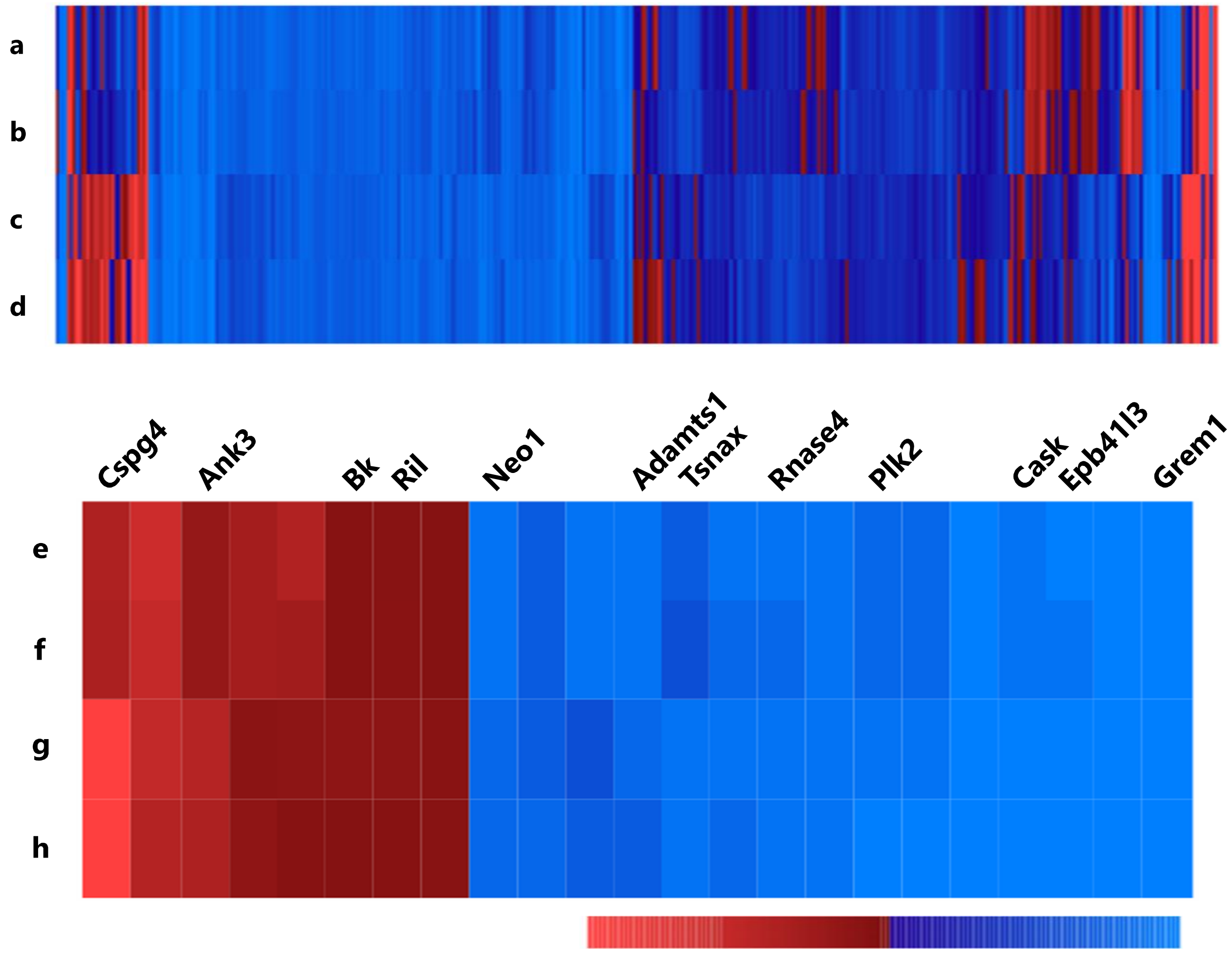

Heatmap representation of relative gene expression including 1042 **(a, b, c, d)** and 23 **(e, f, g, h)** genes with the largest upregulation or downregulation in the metastatic cell populations compared to the non-metastatic population. The brightest red represents maximum upregulation and the brightest blue represents maximum downregulation with the range of intermediate colors presented as the pseudo-color bar under the heatmaps. The majority of changes is downregulation corresponding to dedifferentiation. **(a, e)** The ratio of normalized expression levels in T15 and K2 cells taken from tissue culture. **(b, f)** The ratio of normalized expression levels in A297 and K2 cells taken from tissue culture. **(c, g)** The ratio of normalized expression levels in T15 and K2 cells taken from primary tumors. **(d, h)** The ratio of normalized expression levels in A297 and K2 cells taken from primary tumors. In some cases, the gene expression regulation is different in tissue culture in comparison to the primary tumor. Only genes with a statistically significant change of at least 2.5× are included in **(e, f, g, h)**. Names of genes are taken from the expression chip annotation.

Supplementary Video S4 Time-lapse sequence (lapse interval of 5 min) with alternating phase contrast (left) and green fluorescence images shown as greyscale (middle) of T15 sarcoma cells in the Dunn chemotaxis chamber after some of the cells had been microinjected with EGFP expression construct and allowed for 3 h incubation to produce the EGFP. Overlay with fluorescence images in green is shown in the right part of the panel. The time-lapse acquisition was performed with Kinetic Imaging software and an E1000 microscope (Nikon) equipped with a 10×/0.3 Ph1 objective lens and an Orca ER scientific CCD camera. The field size is 627 × 501 μm. Transfected and control cells are present in the same observation field simplifying their comparison.

Supplementary Video S5 Time-lapse recording of pneumocytes type II (related to Fig. 2a, d) and A549 cells (related to Fig. 2b, e) acquired with lapse interval of 15 min and shown as an overlay. The field size is 322 × 322 μm.

Supplementary Video S6 Time-lapse recording of T15 cells (related to Fig. 3a) acquired with lapse interval of 1 min.

Supplementary Video S7 Time-lapse recording of control A549 cells (related to Fig. 5a, b) acquired with lapse interval of 1 min presenting every 10th frame.

Supplementary Video S8 Time-lapse recording of GDC treated A549 cells acquired with lapse interval of 1 min presenting every 10th frame. Field size and color interpretation as in Fig. 5a.

Supplementary Video S9 Time-lapse recording of GSK treated A549 cells acquired with lapse interval of 1 min presenting every 10th frame. Field size and color interpretation as in Fig. 5a.

Supplementary Table S10

Parameter \ Treatment	CNT	DMSO	NVP	GDC	PD	GSK
Dry-mass doubling time [h]	22.1±1.2	21.1±1.0	23.9±1.6	30.4±1.3**^{1}	23.8±1.7	26.7±1.9*^{2}
Speed [μm h^{-1}]	17.0±1.0	16.7±1.0	16.3±0.9	12.8±1.2**^{3}	18.5±1.2	22.1±2.2
Protrusion mass [pg]	2.3±0.3	2.0±0.3	1.5±0.1	1.7±0.2	1.8±0.1	1.4±0.1
Max mass in the protrusion area [fg]	85.2±2.1	72.5±2.1	77.7±2.5	68.8±2.4*^{4}	88.9±4.3	97.2±3.9
Dynamic polarity [μm]	17.2±0.8	14.9±0.8	18.3±1.2	16.9±1.2*^{5}	16.8±0.8	19.5±1.8
Number of movies	6	5	5	3	4	3
Number of tracked cells	197	128	173	123	199	138
Number of time point measurements	27 510	15 283	36 575	27 012	31 557	25 528

Mean±S.E.M. data from iQPI analysis of A549 responses. The values were calculated from means for individual cells ignoring the level of recordings. S.E.M. stands for standard error of the mean. ***** and ****** mark differences with significant ANOVA P-value. ****^{1}** P<0.02 vs. CNT and P<0.002 vs. DMSO; ***^{2}** P<0.03 vs. DMSO; ****^{3}** P<0.04 vs. CNT and P<0.04 vs. DMSO; ***^{4}** P<0.05 vs. CNT; and ***^{5}** P<0.03 vs. CNT.

Supplementary Figure S11

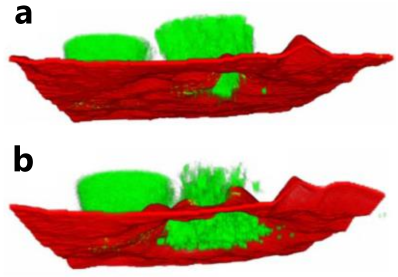

Illustration of the invasion assay with a 3D reconstruction from stacks of wide field images acquired using 60× NA 1.45 Nikon lens. Reconstructed top surface of the monolayer cells is in red. Left hand side greed cancer cells is adherent and the right hand side green cancer cell is shown at early **(a)** and late **(b)** invasion stages. Volume 54 µm × 54 µm × 21 µm.

Supplementary Figure S12

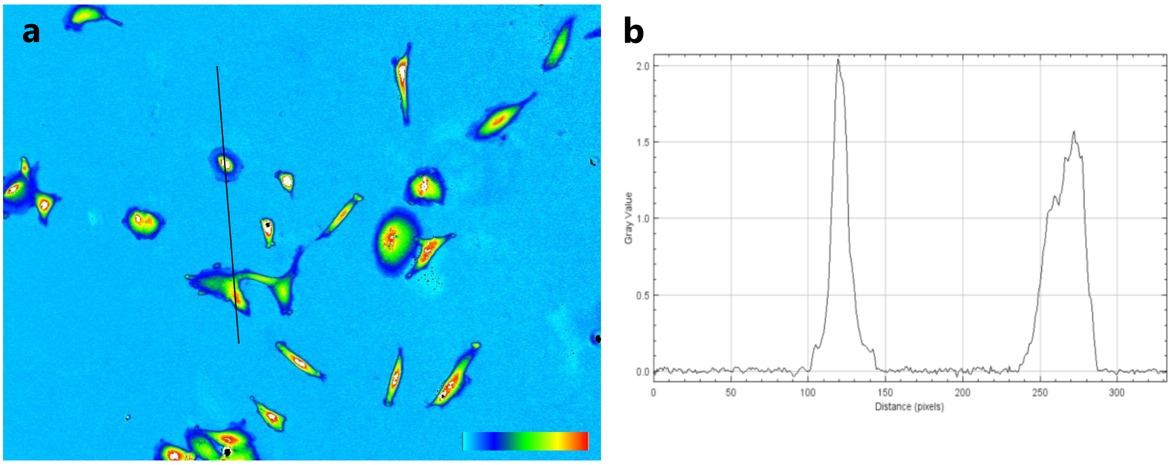

Example of a quantitative phase image **(a)** after subtraction of the reconstructed background related to Fig. 5a. Field size is 634 × 483 µm. Pseudo-color bar represents a range of dry-mass densities from 0 to 0.86 pg µm^{-2}. Profile along the black line is in **b** and illustrates the clean background due to the incoherent light source which allowed thresholding at 0.07 rad which is around 1% of the max cell phase retardations.
