# Supplementary figures and images for: Addressing cancer invasion and cell motility with quantitative light microscopy
Source: Sci Rep. 2022 Jan 31;12:1621. doi: 10.1038/s41598-022-05307-7 (PMC8803927; doi:10.1038/s41598-022-05307-7)

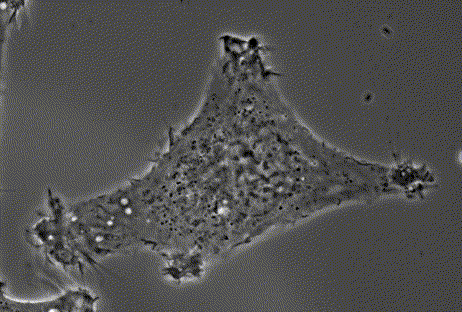

Supplement: Supplementary file 2 — Supplementary Video S1. [file 41598_2022_5307_MOESM2_ESM.zip › DanielZicha_Video_S1.gif]

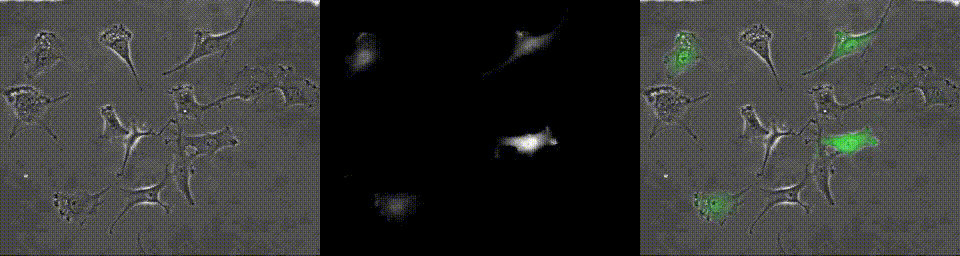

Supplement: Supplementary file 3 — Supplementary Video S4. [file 41598_2022_5307_MOESM3_ESM.zip › DanielZicha_Video_S4.gif]

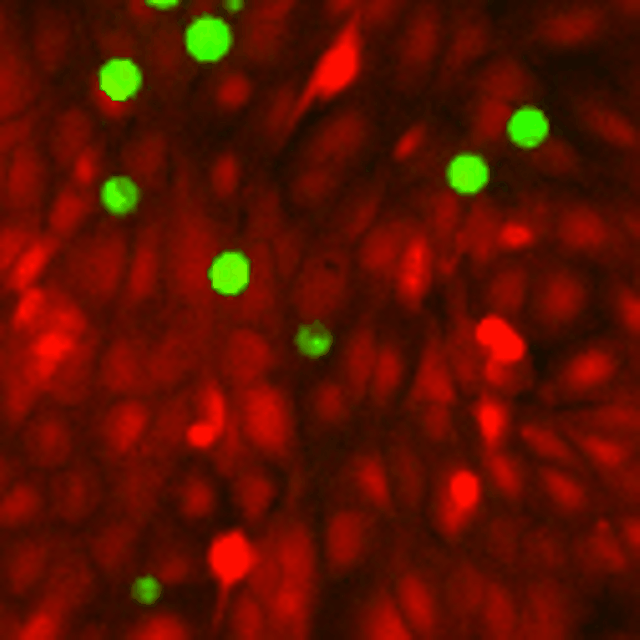

Supplement: Supplementary file 4 — Supplementary Video S5. [file 41598_2022_5307_MOESM4_ESM.zip › DanielZicha_Video_S5.gif]

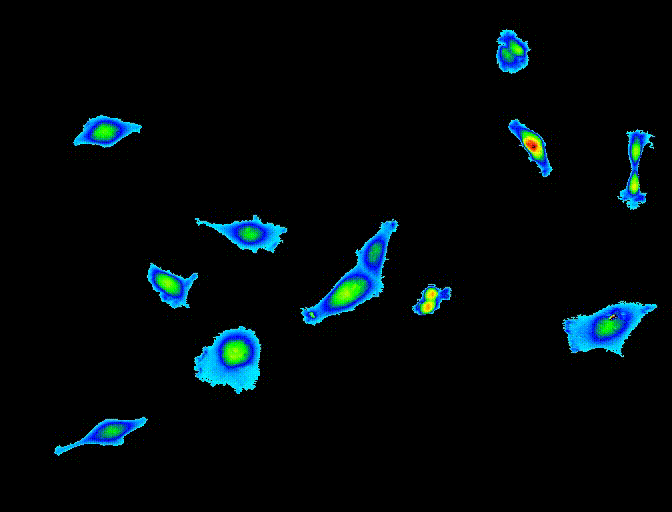

Supplement: Supplementary file 5 — Supplementary Video S6. [file 41598_2022_5307_MOESM5_ESM.zip › DanielZicha_Video_S6.gif]

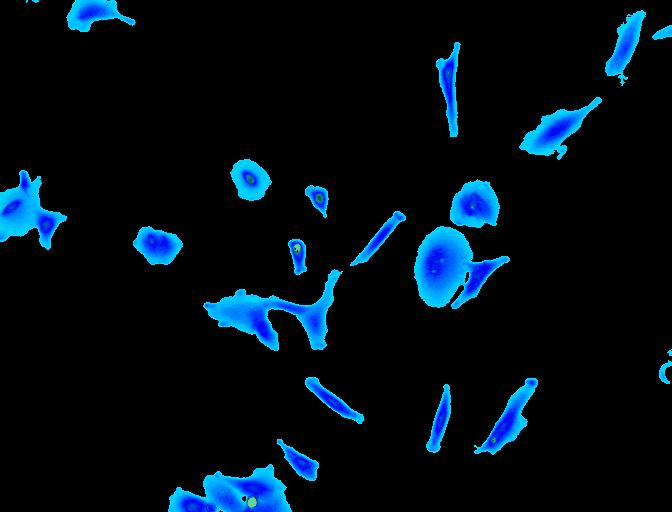

Supplement: Supplementary file 6 — Supplementary Video S7. [file 41598_2022_5307_MOESM6_ESM.zip › DanielZicha_Video_S7.gif]

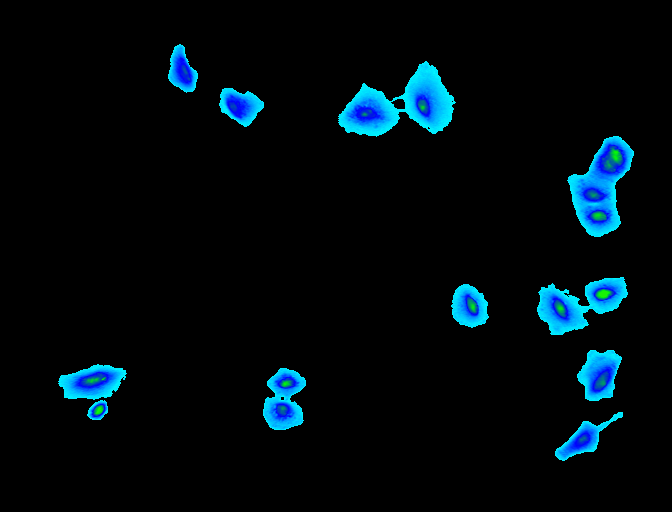

Supplement: Supplementary file 7 — Supplementary Video S8. [file 41598_2022_5307_MOESM7_ESM.zip › DanielZicha_Video_S8.gif]

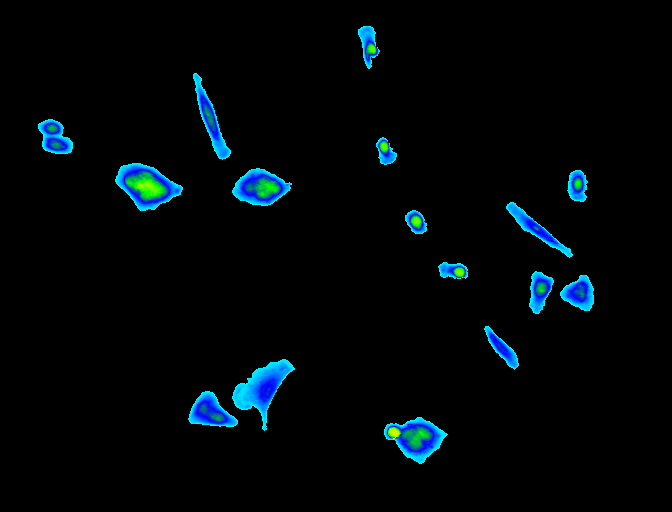

Supplement: Supplementary file 8 — Supplementary Video S9. [file 41598_2022_5307_MOESM8_ESM.zip › DanielZicha_Video_S9.gif]
